# The Leaf Microbiome of *Arabidopsis* Displays Reproducible Dynamics and Patterns throughout the Growing Season

**DOI:** 10.1128/mbio.02825-21

**Published:** 2022-04-14

**Authors:** Juliana Almario, Maryam Mahmoudi, Samuel Kroll, Mathew Agler, Aleksandra Placzek, Alfredo Mari, Eric Kemen

**Affiliations:** a Microbial Interactions in Plant Ecosystems, IMIT/ZMBP, Eberhard Karls University of Tübingen, Tübingen, Germany; b Université Claude Bernard Lyon 1, CNRS, INRA, Villeurbanne, France; c Max Planck Research Group Fungal Biodiversity, Max Planck Institute for Plant Breeding Research, Cologne, Germany; d Plant Microbiosis Group, Institute for Microbiology, Friedrich Schiller University Jena, Jena, Germany; University of Toronto

**Keywords:** leaf microbiome, time dynamics, microbial networks, microbial hubs, community dynamics, core microbial community, hub microbes, microbial communities, persistence, plant-microbe interactions

## Abstract

Leaves are primarily responsible for the plant’s photosynthetic activity. Thus, changes in the leaf microbiota, which includes deleterious and beneficial microbes, can have far-reaching effects on plant fitness and productivity. Identifying the processes and microorganisms that drive these changes over a plant’s lifetime is, therefore, crucial. In this study, we analyzed the temporal dynamics in the leaf microbiome of Arabidopsis thaliana, integrating changes in both composition and microbe-microbe interactions via the study of microbial networks. Field-grown *Arabidopsis* were used to monitor leaf bacterial, fungal and oomycete communities throughout the plant’s natural growing season (extending from November to March) over three consecutive years. Our results revealed the existence of conserved temporal patterns, with microbial communities and networks going through a stabilization phase of decreased diversity and variability at the beginning of the plant’s growing season. Despite a high turnover in these communities, we identified 19 “core” taxa persisting on *Arabidopsis* leaves across time and plant generations. With the hypothesis these microbes could be playing key roles in the structuring of leaf microbial communities, we conducted a time-informed microbial network analysis which showed core taxa are not necessarily highly connected network “hubs,” and “hubs” alternate with time. Our study shows that leaf microbial communities exhibit reproducible dynamics and patterns, suggesting the potential of using our understanding of temporal trajectories in microbial community composition to design experiments aimed at driving these communities toward desired states.

## INTRODUCTION

Leaves are primarily responsible for the plant’s photosynthetic activity and gaseous exchange. Consequently, leaf health and performance have a direct effect on plant growth and fitness (1). Leaves are colonized by a wide range of microbes, including bacteria, archaea, and microeukaryotes such as fungi and oomycetes. While natural openings on leaves such as stomata, hydathodes, or wounds represent entry points for major plant pathogens, they also house commensal and even beneficial microbes (2, 3), leading to plant-protecting effects (4–6). There is increasing interest particularly by plant breeders in microbiota-engineering approaches to promote the growth and health of crops through beneficial microbes (7). In this context, it is essential to understand the processes that shape the composition of leaf microbiota.

There is a level of specificity between plants and their leaf microbiota as studies have repeatedly shown that different plant lineages tend to harbor quantitatively different microbial consortia in their leaves (8), with differences even observed between ecotypes of the same plant species (9). Although it is unclear how plants can selectively recruit certain microbial groups, the soil in which plants grow appears to be an important driver (9, 10). The study of plant microbiota over different developmental stages suggests that as the plant grows, the microbiota becomes more tissue-specific with major differences observed between root and shoot microbiota (11, 12). There is increasing awareness of the fact that plant-associated microbiota are not static but dynamic communities whose members engage in multiple layered interactions, such as mutualism, antagonism, or predation, which change through time under the convergent influence of environmental and host cues and neighboring plants (13). Indeed, leaf microbial communities are constantly exposed to the arrival of new microbes carried by soil, water, and wind and can thus show a high level of stochasticity, i.e., high unpredictability and high variability. Furthermore, leaf communities have been shown to change throughout time and reach different stable states, depending on early (random) events (priority effects) (14). Recent studies have followed the dynamics of microbiome formation in leaves (13–18) and roots (19), but few of them have conducted a cross-kingdom survey, integrating both bacterial and microeukaryotic communities, which means we only have partial views of microbial dynamics in leaves.

Correlation network analyses on the relative abundance of microbial taxa can inform us about potential interactions between community members, albeit with high rates of false-positive and false-negative interactions among the predictions (20). Still, the combination of in-depth analysis of microbial coabundance networks with hypothesis-testing experiments has led to the description of new biological interactions in host-associated microbiomes (21), including plant microbiomes (22). Moreover, the study of microbial networks over time can inform us about the dynamics of these potential interactions and how they relate to changes in the diversity and structure of microbial communities (23). However, such approaches have rarely been applied to investigate how plant-associated microbiome change through the plant’s life.

Given the complexity of leaf microbial communities, assigning ecological roles and ecological importance to individual taxa is extremely challenging. Concepts based on the persistence of a microbe (core taxa) and/or its importance on microbial networks (hubs taxa) have been applied to identify microorganisms playing key roles in leaf communities (22, 24). Although the large majority of leaf microbes show scattered distributions with highly fluctuating occurrences in plant leaves across environments and time, some microorganisms achieve a stable presence in plant populations (25). It is unclear how these “core” microbes are able to systematically colonize the host plant, but their “persistence” could involve recolonization processes (26) or vertical inheritance via seeds (27). The stability of the associations between “core” microbes and the host-plant suggests a high level of adaptation to the leaf niche by microbes. This can involve traits associated with plant colonization and infection, as suggested for leaf-pathogenic Pseudomonas viridiflava (25), but it can also involve the capacity of the microorganism to reshape the leaf microbiota, as part of a “niche construction” strategy. Notably, Agler et al. (22) showed that the inoculation of the leaf-pathogenic oomycete *Albugo* on Arabidopsis thaliana plants translates into decreased microbial diversity on leaves and altered microbiome profiles. The analysis of microbial interaction networks within the leaf microbiome showed *Albugo* acts as a network “hub,” showing the highest level of connections (interactions) with other microbes, which would allow it to influence the structure of the leaf microbiota. Because of its hub characteristics and experimentally proven impact on leaf microbial communities, *Albugo* has been proposed as a “keystone” taxon of the leaf microbiota in *Arabidopsis*. However, it is still unclear whether reshaping the leaf microbiome contributes to persistence of core taxa.

The aim of this study was to analyze the temporal dynamics in the leaf microbiome of Arabidopsis thaliana, integrating both compositional changes and changes in microbe-microbe interactions via the study of microbial networks. Amplicon sequencing was used to follow leaf bacterial, fungal, and oomycete communities in a field experiment throughout the natural growing season of *Arabidopsis*, which, in the Cologne area, extends from November (seedling emergence) to March (beginning of flowering). The experiment was carried out in a common garden over three consecutive years in order to capture long-term dynamics, and four *Arabidopsis* ecotypes were included. Our results reveal seasonal/monthly patterns associated with reproducible changes in particular groups across kingdoms like *Sphingomonadales* and *Actinomycetales* bacteria, *Microbotryales* and *Sporidiobolales* fungi, and *Peronosporales* oomycetes. Despite a high level of stochasticity in microbial colonization of the leaf, we identified 19 taxa that were consistently present (core taxa), including putative pathogenic and beneficial taxa. Between November and February, the diversity and variability of leaf microbial communities decreased, as microbial networks stabilized (changed less) and exhibited decreasing complexity (number of nodes and connections). With the hypothesis that certain microbes play a predominant role in the structuring and stability of these communities, we focused on the identification of microbes having both a persistent presence on *Arabidopsis* leaves (core microbes) and a high connectivity in leaf microbiome networks (hub microbes).

## RESULTS

### The leaf microbiome is highly dynamic.

**FIG 1:**
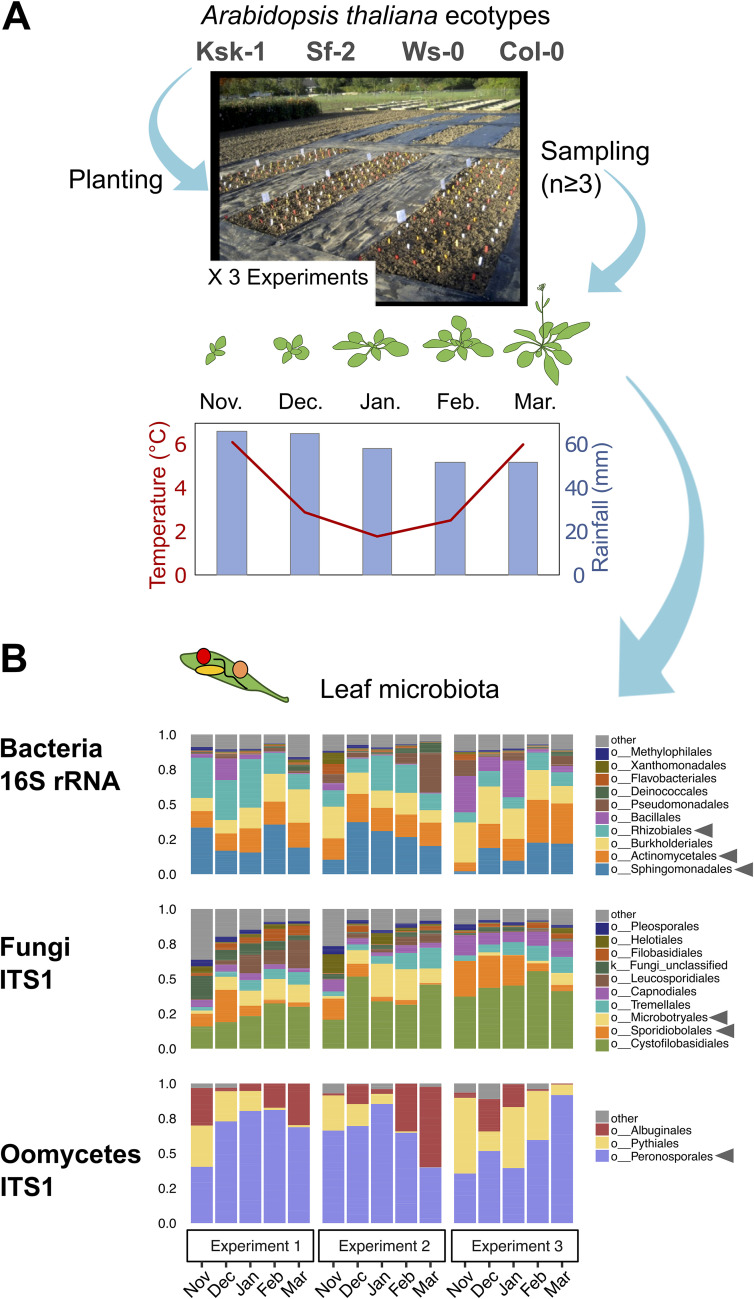
Monitoring leaf microbiome dynamics throughout the natural growing season of *A. thaliana.* (A) Experimental setup. The four global *Arabidopsis* accessions Ws-0, Col-0, Ksk-1, and Sf-2 were planted in a common garden (Max Planck Institute, Cologne, Germany). Every month from November to March, three individual plants per ecotype were collected, and leaf samples were taken for microbiome analysis (destructive sampling). The experiment was repeated three times over the years 2014 to 2015 (experiment 1), 2015 to 2016 (experiment 2), and 2016 to 2017 (experiment 3), with a total number of 206 plant leaf samples analyzed (see Table S1). Average temperature and rainfall during the sampling season are shown. (B) Composition of the leaf microbiome. Microbiome analysis was conducted via Illumina-based amplicon sequencing (Miseq 2 × 300 bases). Taxonomic markers included the bacterial 16S rRNA v5-v7 region, fungal ITS1, and the oomycete ITS1 region. Bar charts show the average relative abundance of the main microbial groups (order level) by months, across three experiments. Arrowheads indicate taxa exhibiting marked seasonal patterns (see Fig. S2).

To study the temporal dynamics of the leaf microbiome, we grew four *A. thaliana* ecotypes in a common garden and surveyed the changes in their leaf microbiome via amplicon-sequencing (bacteria, fungi, and oomycetes). Leaf samples were taken monthly between November and March (5 months), thus covering most of the plant’s growing season over autumn and winter (Fig. 1; see also Table S1 in the supplemental material). To identify the main factors shaping leaf microbial communities, we used multivariate approaches, including nonmetric multidimensional scaling (NMDS; Fig. S1A) and permutational multivariate analysis of variance (ANOVA; Bray-Curtis dissimilarities, *P < *0.05; see Fig. S1B) on the relative abundance of bacterial, fungal and oomycete taxa (operational taxonomic units [OTUs] defined at 97% similarity). These analyses showed a marginal effect of the plant ecotype (2 to 4% explained variance) but an important effect of the time of sampling (32 to 40% explained variance; factors “month,” “experiment,” and their interactions; see Fig. S1B), confirming that leaf microbial communities are highly variable in time (i.e., dynamic). Although variability between experiments was significant (4 to 13% explained variance), the “month” of sampling was an important factor (11 to 15% explained variance; Fig. S1A and B), suggesting the existence of seasonal/monthly patterns in these microbial communities. Such patterns were easily observable when considering changes in the relative abundance of highly abundant microbial orders (Fig. 1). For example, the relative abundances of *Sphingomonadales* and *Actinomycetales* increased throughout the plant’s growing season, while the relative abundance of *Rhizobiales* tended to decrease. As for fungi, the relative abundance of *Microbotryales* increased, while that of *Sporidiobolales* decreased. Interestingly, the relative abundance of Peronosporales oomycetes, which include *A. thaliana*’s pathogen *Hyaloperonospora* spp., increased with time, reaching maximum values at the end of the plant’s growing season (Dunn test, *P < *0.05) (Fig. 1; see also Fig. S2).

10.1128/mBio.02825-21.1 FIG S1 Multivariate analysis on factors structuring leaf microbial communities. (A) Nonmetric multidimensional scaling ordination (NMDS) on Bray-Curtis dissimilarities between samples grouped by “month,” “experiment,” or “ecotype.” (B) Circles depict the percentage of variance explained by factors “month,” “experiment,” and “ecotype,” connecting lines depict the percentage of variance explained by interactions between factors. A PerMANOVA analysis on Bray-Curtis distances was conducted using the Adonis function in Vegan. Only significant effects are shown (permutations, 10,000; *P < *0.05; explanatory categorical variables: experiment × month × ecotype). Download FIG S1, TIF file, 2.8 MB. Copyright © 2022 Almario et al. 2022 Almario et al. https://creativecommons.org/licenses/by/4.0/ This content is distributed under the terms of the Creative Commons Attribution 4.0 International license.

10.1128/mBio.02825-21.2 FIG S2 Temporal changes in high-abundance microbial taxa colonizing *A. thaliana*’s leaves. Boxplots show the relative abundance of the bacterial (green), fungal (orange), and oomycete (blue) orders in single samples aggregated by “month.” Whiskers depict the dispersion of the data (1.5 × interquartile range), and different letters indicate significant differences between months (Dunn test, *P < *0.05). Download FIG S2, TIF file, 1 MB. Copyright © 2022 Almario et al. 2022 Almario et al. https://creativecommons.org/licenses/by/4.0/ This content is distributed under the terms of the Creative Commons Attribution 4.0 International license.

10.1128/mBio.02825-21.5 TABLE S1 Experimental setup. Numbers indicate sampled plants by condition. Download Table S1, XLSX file, 0.01 MB. Copyright © 2022 Almario et al. 2022 Almario et al. https://creativecommons.org/licenses/by/4.0/ This content is distributed under the terms of the Creative Commons Attribution 4.0 International license.

### Persistent (core) taxa in the leaf microbiome.

**FIG 2:**
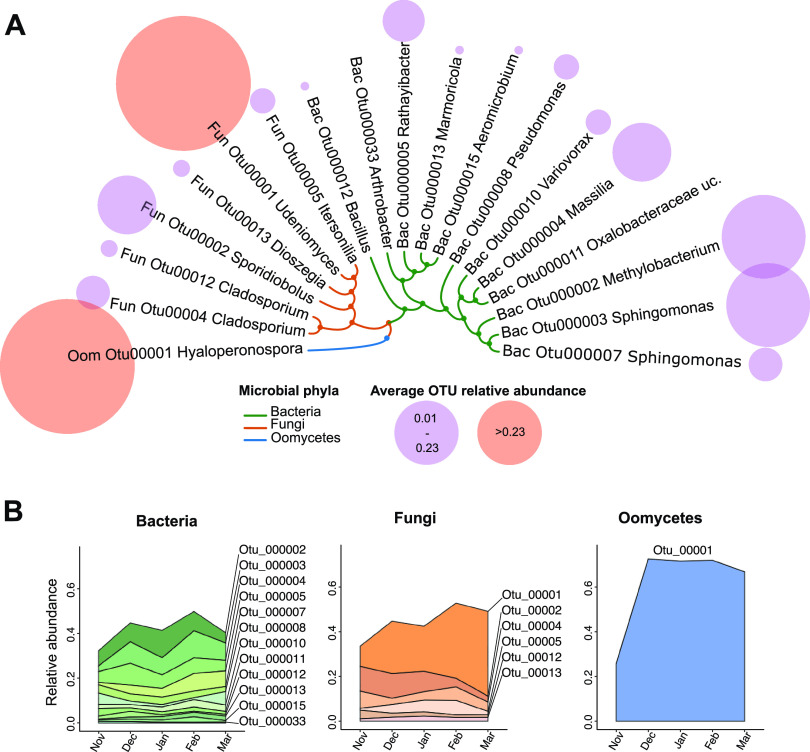
Persistent core members of the *Arabidopsis* leaf microbiome. (A) Core taxa were identified as OTUs showing high occurrence results (≥95% for fungi and oomycete, ≥98% for bacteria) in each of the three experiments. Bubbles depict the average relative abundance of each core OTU, per sample. The dendrogram depicts taxonomical distances between OTUs (hierarchical clustering on Gower distances from OTU taxonomy). (B) Changes in the relative abundance of core taxa over time (month averages; *n* > 38 samples per month).

We aimed to identify microbial groups showing a persistent presence throughout the plant’s life, hypothesizing that they might play important roles in plant-microbe and microbe-microbe interactions within the microbiome. Highly persistent microbes (≥95% sample occurrence for fungi and oomycete, ≥98% for bacteria) varied considerably between experiments, with only 19 of 67 OTUs (28%) showing robust patterns across experiments and ecotypes (see Fig. S3 and Table S2). Notably, these persistent core taxa (1 oomycete, 6 fungus, and 12 bacterial OTUs) included known *Arabidopsis* pathogens like the obligate biotrophic oomycete *Hyaloperonospora* sp. (Otu00001), as well as bacterial taxa known to colonize *Arabidopsis* leaves, including *Sphingomonas* spp. (OTUs), *Methylobacterium* sp. (Otu000002), and *Variovorax* (Otu000010). Persistent fungal taxa included two ascomycetes (C*ladosporium* spp. Otu00004 and Otu00012) and four basidiomycete yeast (*Dioszegia* sp. Otu00013, *Itersonilia* sp. Otu00005, *Sporidiobolus* sp. Otu00002, and *Udeniomyces* sp. Otu00001) (Fig. 2A; see also Table S2). The relative abundances of these core taxa changed throughout the plant’s growing season, reaching a maximum in February, where it represented as much as 49, 52, and 71% of the bacterial, fungal, and oomycete communities, respectively (Fig. 2B). These results indicate that despite the highly dynamic and stochastic nature of the leaf microbiome, a limited number of microbes—only 19 of 3,058 OTUs (0.62%)—consistently cocolonize plant leaves. This suggests a high degree of adaptation to this niche but also frequent interactions with one another.

**FIG 3:**
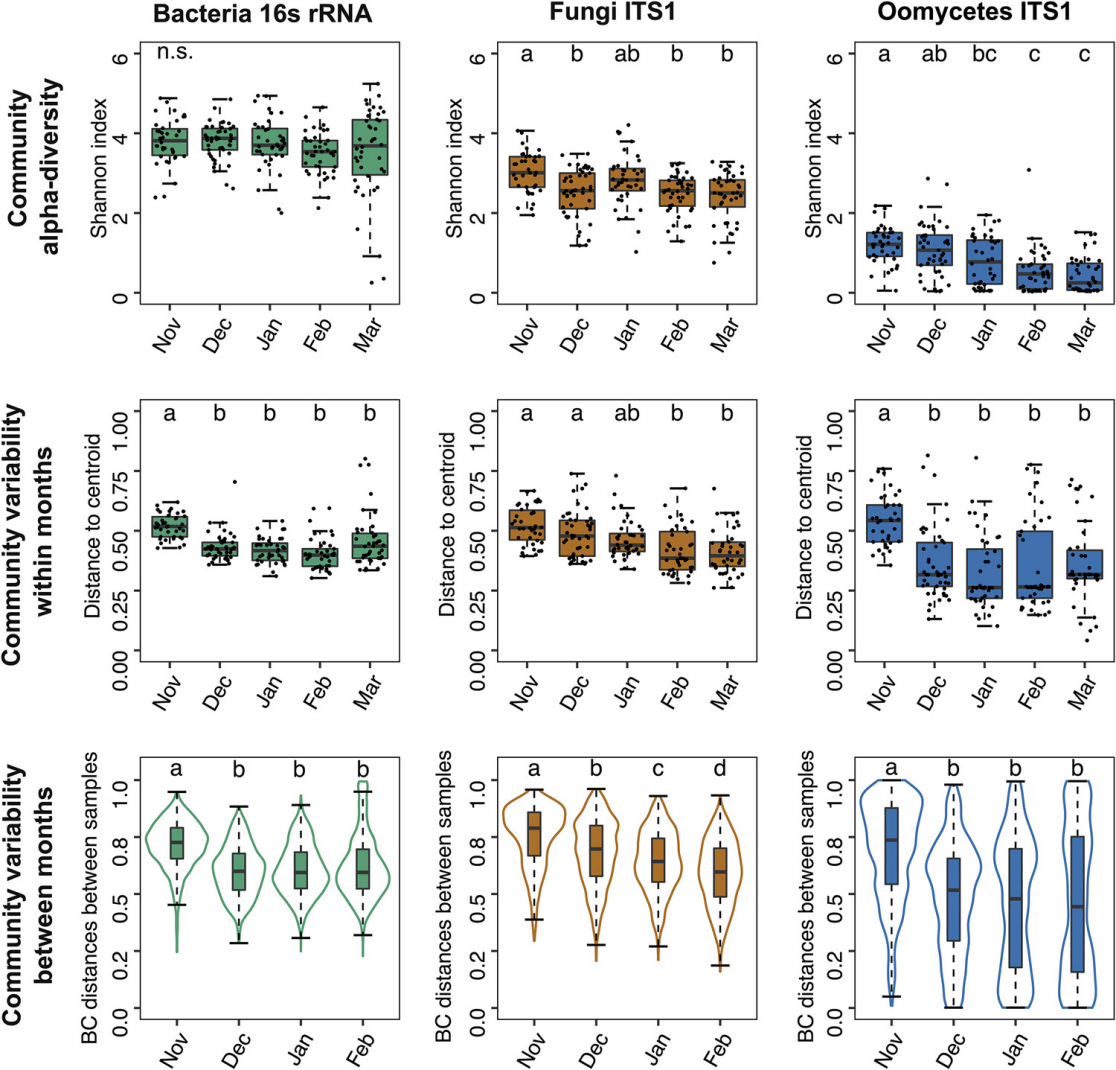
Changes in alpha diversity and variability in leaf microbial communities over time. The alpha diversity (Shannon’s H index), within-month variability (distance to the group centroid; beta-dispersion), and between-month variability (Bray-Curtis distances between samples from consecutive months) in bacterial, fungal, and oomycete communities are shown. Each plot shows combined data from the three experiments, with *n* > 38 samples per month. Dots represent individual samples, whiskers depict the dispersion of the data (1.5 × interquartile range), and different letters indicate significant differences between groups (Dunn test, *P < *0.05). Single BC distances between samples are not shown because of the high number of comparisons (>700).

10.1128/mBio.02825-21.3 FIG S3 Identification of persistent core microbial taxa. Persistent core taxa were identified as OTUs showing high occurrence (≥95% for fungi and oomycete, ≥98% for bacteria) in each of the three experiments analyzed. Purple inner rings depict OTU occurrence within each year, while the outer black ring denotes OTUs identified as “core” (names highlighted in boldface) (see Fig. 3). Download FIG S3, TIF file, 1.2 MB. Copyright © 2022 Almario et al. 2022 Almario et al. https://creativecommons.org/licenses/by/4.0/ This content is distributed under the terms of the Creative Commons Attribution 4.0 International license.

10.1128/mBio.02825-21.6 TABLE S2 Bacterial core OTUs. Green genes and Silva taxonomies of bacterial core OTUs shown in Fig. 2 and Fig. S3. Download Table S2, XLSX file, 0.01 MB. Copyright © 2022 Almario et al. 2022 Almario et al. https://creativecommons.org/licenses/by/4.0/ This content is distributed under the terms of the Creative Commons Attribution 4.0 International license.

Diversity and variability of the leaf microbiome decrease throughout the plant’s growing season as communities stabilize. With the hypothesis that leaf-associated microbial communities become increasingly stable throughout the plant’s growing season, we analyzed their dynamics in terms of alpha diversity (number of taxa in the community), within-month variability (plant-to-plant differences in community composition), and variability between consecutive months (month-to-month differences in community composition). While the bacterial alpha diversity (Shannon’s H index) remained unchanged, the fungal and oomycete alpha diversity decreased with significant differences observed between November and the last 2 months, February and March (Dunn test, *P < *0.05) (Fig. 3). A similar trend was observed for within-month variability (sample distance to the group centroid), as variability of bacterial and fungal communities decreased progressively from November to February (Dunn test, *P < *0.05) (Fig. 3). Similarly, a progressive decrease in between-month variability (sample-to-sample distances between consecutive months) was observed for bacterial and fungal communities (Dunn test, *P < *0.05; Fig. 3). Oomycete communities exhibited similar trends, but the dynamics were less pronounced due to higher data variability. Together, these results suggest that throughout the plant’s growing season, leaf microbial communities become progressively less diverse, more similar between plant individuals, and less variable in time. This suggests leaf communities go through a consolidation and stabilization phase from November to February.

### Interaction networks within the leaf microbiome stabilize over time.

**FIG 4:**
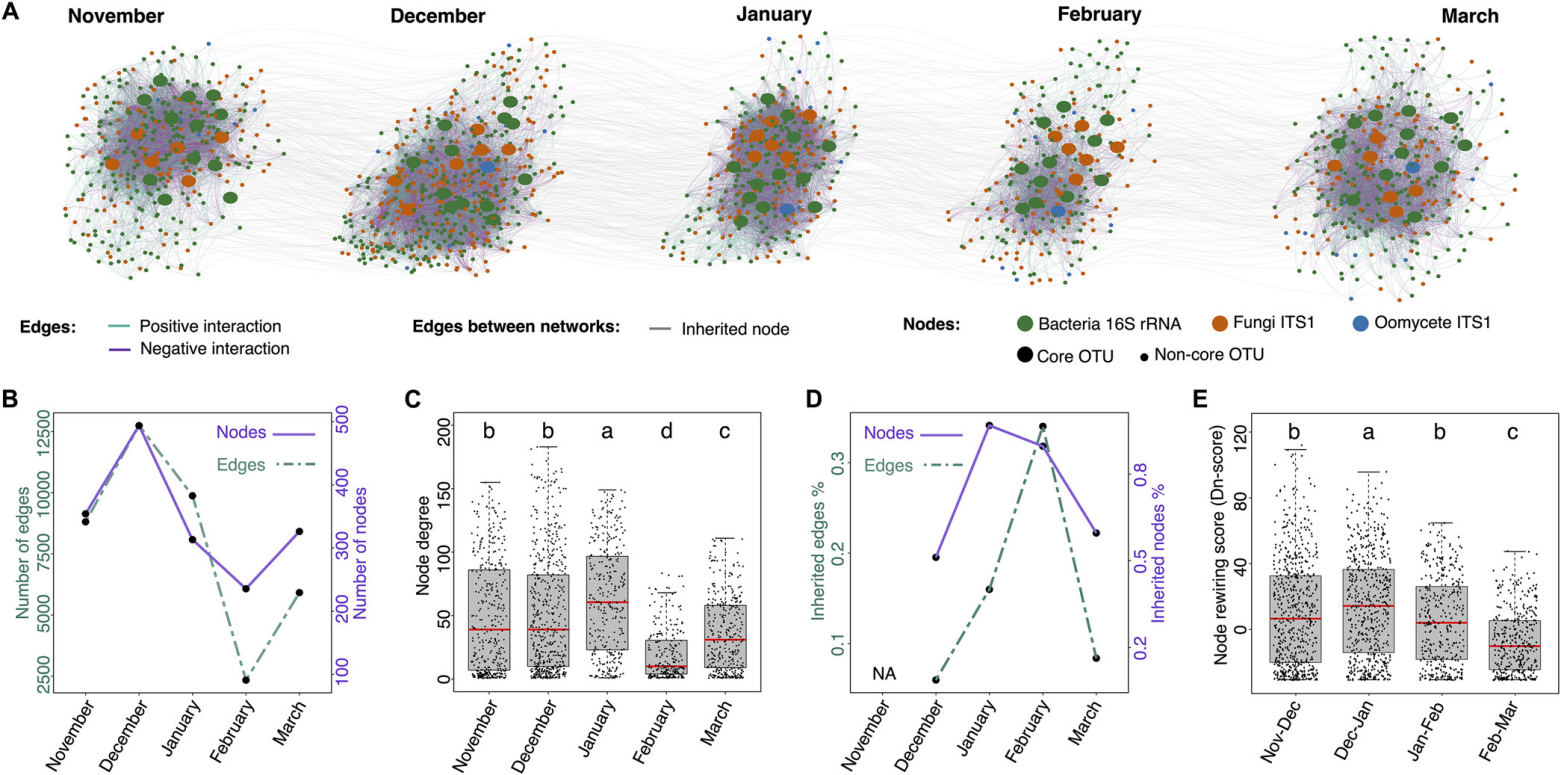
Changes in phyllosphere microbial interaction networks throughout *A. thaliana’s* growing season. (A) Data from the three experiments were aggregated to reconstruct co-abundance networks for each time point (month) using the SparCC algorithm. Nodes (dots) represent OTUs; edges (colored lines) depict potential positive and negative interactions between OTUs (connections). Nodes from core microbes are indicated. Gray lines connecting networks show nodes conserved in networks from 1 month to the next (inherited nodes). (B) Number of nodes and edges in each month network. (C) Percentage of nodes and edges in a given month network which are inherited from (shared with) the previous month network. (D) Percentage of edges inherited for a given inherited node. (E) Node degree, i.e., number of edges per node in each month network. (F) Node-rewiring score (Dn-score) calculated in DyNet. For each node, its connected neighbors are compared between two networks (consecutive months) and the changes (rewiring) are quantified. Points represent rewiring scores from single nodes, high values indicate important changes in the node’s connections between the compared networks. Different letters indicate significant differences between conditions (Dunn test, *P < *0.05).

Microbial networks computed from correlation of species abundances, are used to infer potential interactions between microbes within a community. To determine if/how leaf microbial networks changed over time, we used taxa abundance data from each time point (month) to generate five “month” networks (Fig. 4A). Because the data were highly sparse (53% sparsity), the SparCC algorithm (optimized for sparse data) was used for network calculation (28). The five networks differed in terms of general characteristics such as the number of nodes (number of taxa) and edges (correlations between taxa; syn. connections) with no clear pattern, except for the month of “February,” which had both the lowest number of nodes and the lowest number of edges (Fig. 4B). Similarly, the nodes of this network had the lowest number of interactions (node degree), going from 70 on average in January to only 10 on average in February (Fig. 4B and C; Dunn test, *P < *0.05). This confirmed that microbial networks indeed changed throughout the plant’s growing season and suggested major restructuring events around the month of February, when the network exhibited minimal complexity.

With the hypothesis that these changes were associated with an increased stability of the network’s structure, we compared networks from consecutive months, recording similarities (inherited nodes/edges) and differences (node rewiring events) between them. Inherited nodes/edges were defined as those shared between consecutive months. The percentage of inherited nodes per network increased from 51% in December to 89% in January and 82% February, meaning the large majority (82%) of the nodes in the February network were already present in the January network (Fig. 4D). A similar trend was observed for the number of inherited edges, doubling from December (6%) to January (16%) and February (34%). To quantify changes between networks, taking into account the nodes and their connections, we calculated a node-rewiring score for each node in the network. This score reflects the changes in a node’s connections between the compared networks (Dn-score in DyNet) (29). This analysis revealed that differences between networks tended to decrease through time, with minimum rewiring events between the months of February and March (Dunn test, *P < *0.05) (Fig. 4E). These results suggest that throughout the beginning of the season (November to February) leaf microbial networks go through a stabilization phase, during which month-to-month changes tend to diminish (increasing numbers of shared nodes and edges, and decreasing node rewiring) as networks exhibit lowering complexity (lower numbers of nodes, edges, and connections), reaching minimum levels in February.

### Identifying hubs among core microbes in *Arabidopsis* leaf microbiome.

**FIG 5:**
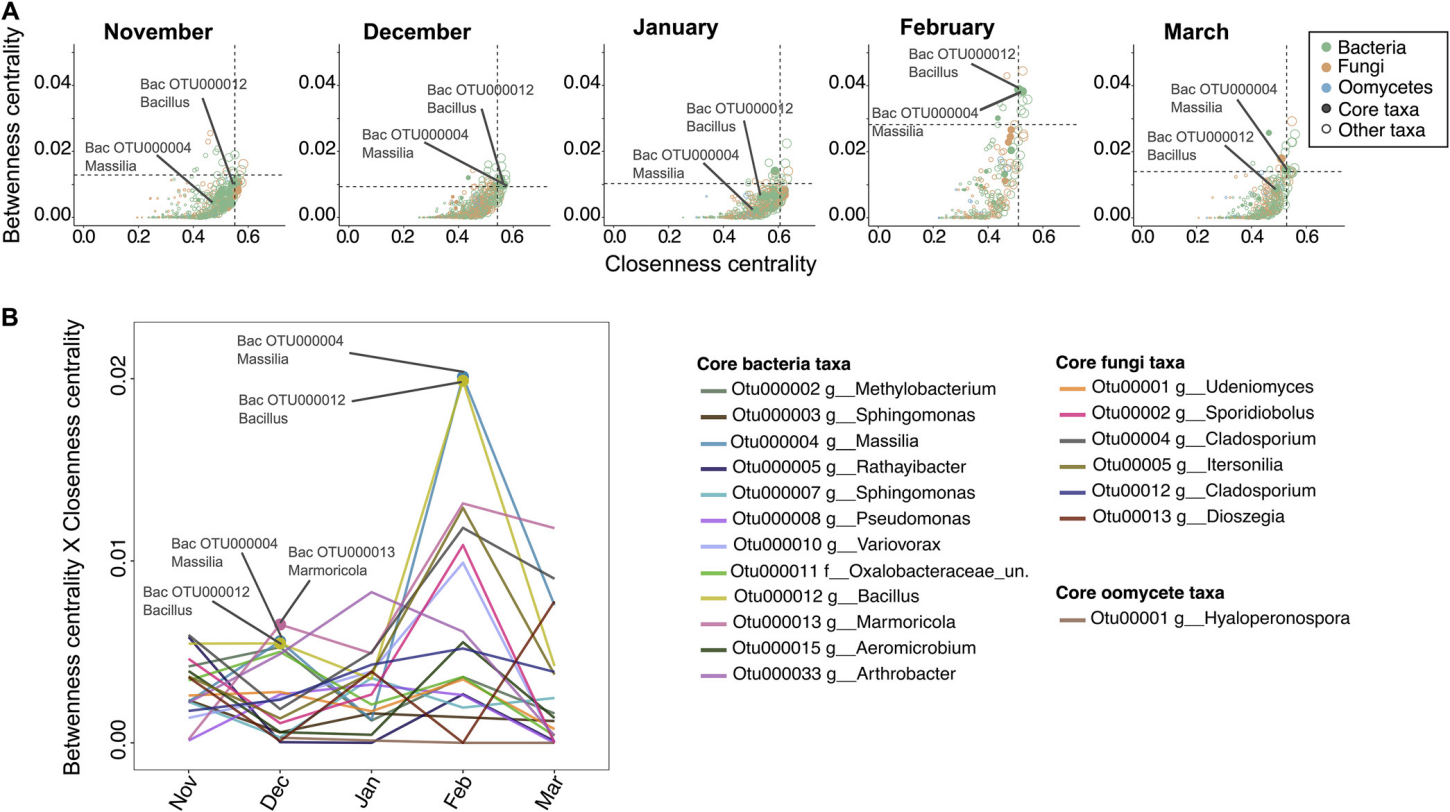
Identification of microbial hubs within *A. thaliana*’s core leaf microbiome. The correlation networks calculated with SparCC (Fig. 3) were used to identify microbial hubs as nodes with high betweenness centrality (i.e., the fraction of shortest paths passing through the given node) and high closeness-centrality (i.e., the average shortest distance from the given node to other nodes). (A) Values for single taxa, with dotted lines indicating the top 5% values. Circles are colored based on microbial phyla. Circle sizes depict the node’s degree. Closed circles indicate taxa identified as part of the core leaf microbiome. Two core OTUs (12 and 4) are annotated. (B) Changes in the connectivity of core taxa. The product of “betweenness centrality × closeness centrality” was used to depict monthly changes in the connectivity of core OTUs. Hub taxa are indicated.

Time-based microbial networks were analyzed to determine whether potential “keystone”’ microbes (i.e., hubs—taxa with high betweenness and high closeness centrality) in the leaf microbiome were also highly persistent core microbes. Connectivity analysis on individual month networks revealed few taxa exhibiting hub characteristics (4 to 10 OTUs, 1 to 3% of network OTU nodes) and a high turn-over between months, with no taxon systematically identified as hub in every month network (Fig. 5A; see also Table S1). Among the 19 “core” taxa identified previously (Fig. 2), only three bacterial OTUs, i.e., *Bacillus* OTU00012, *Massilia* OTU00004, and *Marmoricola* OTU000013, could be identified as hubs exhibiting high network connectivity in the months of December and February (Fig. 5B; see also Table S3).

10.1128/mBio.02825-21.7 TABLE S3 Hub analysis. A comparison of microbial hubs identified via SparCC or Co-Net network analysis is shown. For each month network, microbial hubs were identified as top 5% OTUs showing maximum betweenness centrality and closeness centrality scores. Core microbial taxa (see Fig. 2) identified as hubs by either method are highlighted in orange. Core microbial taxa (see Fig. 2) identified as hubs by both methods are highlighted in yellow. Download Table S3, XLSX file, 0.01 MB. Copyright © 2022 Almario et al. 2022 Almario et al. https://creativecommons.org/licenses/by/4.0/ This content is distributed under the terms of the Creative Commons Attribution 4.0 International license.

As hub identification is highly dependent on network calculation approaches, we repeated these analyses on Spearman-based correlation networks calculated in Co-Net (see Fig. S4) with partially similar results. Approximately a third of the OTUs identified as hubs in the SparCC networks were also identified as hubs in the Spearman-based networks (see Table S1). Notably, this also included *Bacillus* OTU00012 and *Massilia* OTU00004. Taken together, these results indicate that, with the exception of one *Massilia* and one *Bacillus* lineage, “core” taxa in the *Arabidopsis* leaf microbiome are not major network hubs and that network hub microbes change over time.

10.1128/mBio.02825-21.4 FIG S4 Identification of microbial hubs within the leaf microbiome of *A. thaliana* (Co-Net-based networks). (A) Co-Net-based month networks were used to identify microbial hubs as nodes with high betweenness centrality (i.e., the fraction of shortest paths passing through the given node) and high closeness centrality (i.e., the average shortest distance from the given node to other nodes). In each graph, dotted lines indicate the top 5% values. Circles are colored based on microbial phyla. Circle sizes depict de node’s degree. Closed circles indicate taxa identified as part of the core leaf microbiome. Two core OTUs (12 and 4) are highlighted. (B) Changes in the connectivity of core taxa. The product of “betweenness centrality × closeness centrality” was used to depict monthly changes in the connectivity of core OTUs. Identified hub taxa (panel A) are indicated. Download FIG S4, TIF file, 2.6 MB. Copyright © 2022 Almario et al. 2022 Almario et al. https://creativecommons.org/licenses/by/4.0/ This content is distributed under the terms of the Creative Commons Attribution 4.0 International license.

## DISCUSSION

The phyllosphere is a complex microbial habitat due to its direct exposure to a range of abiotic factors—light, humidity, and temperature—that can alter the leaf environment within minutes, hours, or days. Furthermore, leaf microbial communities are directly exposed to the arrival of new microbes disseminated by soil particles, water, and wind (30). In this context, key ecological questions are still unanswered: what is the relative importance of environmental filtering versus biotic interactions in shaping community structures and what is the impact of stochasticity (31)? Our limited understanding of the processes behind colonization of leaf surfaces by microbes and their assembly and persistence thereon under field conditions constitutes a major drawback for the agricultural usage of plant-beneficial microbes (32). To address these fundamental questions, we have conducted a long-term experiment to follow month-to-month changes in the composition of the *Arabidopsis* leaf microbiome during its natural growing season, which, in the Cologne area, extends from November (seedling emergence) to March (beginning of flowering).

As expected for dynamic ecological systems (33), bacterial, fungal, and oomycete leaf-associated communities were highly stochastic, with factors such as the sampling time and the plant ecotype explaining only half of the variability observed (see Fig. S1). Despite high between-experiment variability, robust differences between months were observable for some microbial groups known to be relevant for plant-growth like *Peronosporales* oomycetes (see Fig. S2). *Hyaloperonospora*, the causal agent of downy mildew, was by far the most abundant *Peronosporales* in *Arabidopsis* leaves, as it has been described for various geographic locations elsewhere (26). Although our sampled plants exhibited no downy mildew disease symptoms at any time throughout the field experiments, the relative abundance of *Peronosporales* increased throughout the growing season reaching maximum values in March. This is in agreement with disease dynamics of downy mildew in *Brassicaceae* known to be favored by cold wet weather and could indicate that the pathogenic pressure on the plant increases over the early growing, which takes place in winter for *Arabidopsis* populations of the Cologne area.

The analysis of community dynamics indicates that from November to February leaf microbial communities go through a stabilization phase becoming less diverse and less variable, which results in microbial networks of decreased complexity (Fig. 3 and 4). This is likely associated with the fact that core microbes become increasingly dominant throughout the season (Fig. 3B) but contrasts with previous studies showing higher diversity in *Arabidopsis*’ bacterial microbiome in spring (34). Seasonal dynamics have been described in microbiome associated with plants (17, 18, 35) and animals (36–39) and are thought to be driven partly by environmental cues and perturbations. By monitoring the *Arabidopsis* bacterial leaf microbiome under controlled conditions, Maignien et al. similarly showed that leaf communities become increasingly similar as the microbiome “matures” over time (14). However, the fact that in our study microbiome dynamics mirror decreases in temperature and rainfall associated with winter (Fig. 1) leads us to hypothesize that climatic conditions might be also driving the observed leaf microbiome dynamics, maybe via the selection of cold-resistant microorganisms. Indeed, a strong “winter effect” on microbial communities has been observed in a diversity of environments, including the bee’s gut (39), lake water (40), and air (41). We hypothesize that winter conditions might apply a strong selective filter causing leaf microbial communities to reduce in complexity. Longer experiments are needed to determine whether different dynamics would be observed at later stages, e.g., during spring.

Microbes with a stable presence in *Arabidopsis* leaves (core taxa; Fig. 2) accounted for only 0.62% of all detected leaf-taxa, indicating a high turnover in leaf microbial communities. Interestingly, most microbes identified as “core” in one experiment were not identified as “core” in subsequent experiments, suggesting that most dominant lineages change from year to year. This is in accordance with observations that leaf microbiomes are strongly structured by priority effects during early colonization events, meaning that communities can be alternatively dominated by different microorganisms or core taxa (14). In our study, core taxa included putative plant pathogens like *Hyaloperonospora* and *Cladosporium* (42, 43) but also plant beneficial microorganisms such as *Sphingomonas* and *Variovorax*, which could explain the asymptomatic state of the sampled plants. Leaf-inhabiting *Sphingomonas* bacteria have been shown to protect *Arabidopsis* from bacterial pathogens (4) and are hypothesized to participate in plant disease resistance against root fungal pathogens. *Variovorax* strains have been shown to modulate plant hormonal balance by degrading auxins, thus promoting plant growth under stress conditions (44). However, not only bacteria have been reported to interfere with plant hormone levels; there have been reports of yeasts on *A. thaliana* capable of producing auxin-like indolic compounds (45). We have identified four basidiomycete yeast taxa (*Udeniomyces*, *Sporidiobolus*, *Itersonilia*, and *Dioszegia*) as systematic colonizers of *Arabidopsis* leaves. Although little is known about the associations between these yeasts and *Arabidopsis*, a recent study on a leaf basidiomycete yeast (*Moesziomyces bullatus*) suggests they can play important roles in plant protection by antagonizing pathogenic oomycetes through secretion of protein effectors (46). While previous studies have reported on the prevalence of some of the identified core taxa on *Arabidopsis*’ leaves (12, 22, 47), we show here that these associations persist throughout the plant’s life and between plant generations, suggesting some level of microbial adaptation to the leaf niche or even possible coevolution between core microbes, as well as with the host plant. Future isolation/reinoculation experiments will aim at understanding the ecological role of these microbes in *Arabidopsis* leaves.

Microbe-microbe interactions participate in the structuring of microbial communities, with certain microbes—hub and keystone microbes—playing central roles (48). We hypothesized that high connectivity within leaf microbial networks might explain the persistence of the identified core taxa. However, in contrast to our hypothesis, the connectivity level (hubness) of individual core taxa was highly variable from month to month, with no taxon maintaining high connectivity levels throughout the entirety of the growing season (Fig. 5). This indicates that high connectivity is not a prerequisite for high prevalence in the leaf microbiome as core taxa are not necessarily network hubs (19). Nevertheless, two microbes among the leaf core taxa within the *Bacillus* and *Massilia* lineages deviated from this rule and were identified as hubs. Interestingly, in the month of February when leaf microbial communities displayed the lowest levels of complexity, both *Bacillus* and *Massilia* reached maximum connectivity levels within leaf microbial networks (Fig. 5), while their relative abundances on leaves remained stable (Fig. 2). It is tempting to speculate that there might be a functional link between these hubs and community stability. Indeed, it has been shown that highly connected microbes can be good predictors of the stability of microbial communities (49). In the future, experimental evidence will be needed to improve predictions and to determine whether (and how) hub removal affects the stability of microbial communities over time.

### Conclusions.

Taken together, our results show that, despite a high level of stochasticity, leaf microbial communities exhibit detectable time patterns with stable and unstable components. This study opens a new field of research on time-informed community dynamics in natural host-associated microbiomes. In the long term, these types of studies could make it possible to model and predict microbial community dynamics. Understanding these processes could allow us to design experiments aimed at driving microbial communities toward desired states.

## MATERIALS AND METHODS

### Common garden experiment.

To study the temporal dynamics of *A. thaliana*’s leaf microbiome, we conducted a common garden experiment wherein *A. thaliana* plants were sampled every month from November to March, covering the plant’s natural growing season, including the vegetative and early reproductive growth phases (Fig. 1). The experiment was conducted as described in Agler et al. (22). Briefly, surface-sterilized seeds were germinated on Jiffy pellets for 10 days under greenhouse conditions before transfer to the field. To take into account host genetic variability, four Arabidopsis thaliana ecotypes covering different geographic origins were used (Ws-0 [Wassilewskija, Russia], Col-0 [Columbia, USA], Ksk-1 [Keswick, UK], and Sf-2 [San Feliu, Spain]), using the same seed batch for the three experiments. The field was divided into nine experimental plots which were planted with ten plants per ecotype, in a randomized setup. At each sampling point, whole leaf samples were taken from two to four randomly selected plants per ecotype. The whole experiment was repeated three times in 2014 to 2015, 2015 to 2016, and 2016 to 2017. The field is located at the Max Planck Institute for Plant Breeding Research (Cologne, Germany) (see Table S1).

### DNA extraction and amplicon sequencing.

Samples were processed exactly as described in Agler et al. (22). Briefly, whole-leaf samples were crushed and used for phenol-chloroform-based DNA extraction. The obtained DNA was used for two-step PCR amplification of the V5-V7 region of the bacterial 16S rRNA (primers B799F/B1194R), the fungal ITS1 region (primers ITS1F/ITS2), and the oomycete ITS1 region (primers ITS1O/5.8s-O-R). Blocking oligonucleotides were used to reduce plant DNA amplification (50). Purified PCR products were pooled in equimolar amounts before sequencing on three Illumina MiSeq runs (2 × 300-bp reads) with 10% PhiX control. Primers targeting the oomycete ITS1 region also produced “non-oomycete” reads but at a very marginal level (3%).

### Amplicon sequencing data analysis.

Amplicon sequencing data were processed in Mothur (51) as described in Karasov et al. (25). Single-end reads were paired (*make.contigs* command), and paired reads with more than 5 bases overlap between the forward and reverse reads were kept. Only 100 to 600 bases long reads were retained (*screen.seqs*). Chimeras were checked using Uchime in Mothur with more abundant sequences as reference (*chimera.uchime*, abskew = 1.9). Sequences were clustered into OTUs at the 97% similarity threshold using the VSEARCH program in Mothur (*cluster*, dgc method). Individual sequences were taxonomically classified using the rdp classifier method (*classify.seqs*, consensus confidence threshold set to 80) and the greengenes database (13_8 release) for 16S rRNA data, the UNITE_public database (v12_2017) for fungal ITS1, and Pr2 (v4.10.0) for oomycete ITS1. The PhiX genome was included in each of the databases to improve the detection of remaining PhiX reads. Each OTU was then taxonomically classified (*classify.otu*, consensus confidence threshold set to 66); OTUs with unknown taxonomy at the kingdom level were removed, as were low-abundance OTUs (<50 reads, *split.abund*). This last step removed extreme low abundance (<0.0001%)/low occurrence (<0.48%) OTUs. The taxonomy of bacterial OTUs of interest was further verified using the silva database (v1.38; SINA Aligner). This allowed us to classify bacterial OTU00004 as *Massilia* sp.

Sample alpha-diversity analysis was conducted on OTU abundance tables using Shannon’s H diversity index (estimate_richness function in phyloseq package). Data normality was checked (Shapiro-Wilk’s test), and means were compared using a nonparametric multivariate test (Dunn’s test, Bonferroni-corrected adjusted *P* value [*P*_adj_] <0.05). Beta-diversity analyses were conducted on transformed [log_10_(*x *+* *1)] OTU relative abundance tables. Bray-Curtis dissimilarities between samples were computed and used for nonmetric multidimensional scaling ordination (NMDS, function “ordinate”; Phyloseq package). A PerMANOVA analysis on Bray-Curtis dissimilarities was conducted to identify the main factors influencing the structure of the leaf microbiome (Adonis, Vegan package, 10,000 permutations, *P < *0.05, explanatory categorical variables: experiment × month × ecotype). A beta-dispersion analysis on Bray-Curtis dissimilarities was conducted to compare sample-to-sample variation within each month of sampling (multivariate homogeneity of group dispersion analysis, “betadisper”; Vegan package). Differences between conditions were tested using a nonparametric multivariate test (Dunn’s test, Bonferroni corrected, *P*_adj_ < 0.05). All analyses were conducted in R 3.6.1.

### Identification of a core leaf microbiome in *A. thaliana*.

Core taxa were identified as OTUs showing high occurrence over time (≥95% for fungi and oomycete, ≥98% for bacteria) in each of the three experiments analyzed. A higher cutoff was used for bacteria (98% occurrence) since they exhibited a higher average occurrence compared to fungi and oomycetes. The taxonomical classification of core OTUs was used to compute pairwise dissimilarities (distances) between OTUs (“daisy” function, Cluster package in R, Gower’s distance) which were used for hierarchical clustering (“hclust” function, Cluster package in R). The obtained dendrogram was modified in the browser version of iTOL (v5.5.1) (52).

### Network analysis.

Bacteria, fungi and oomycete OTU tables were merged and used for correlations calculation using either the Spearman correlation coefficient in Co-Net (53) or the SparCC algorithm (20), which relies on Aitchison’s log-ratio analysis and is designed to deal with compositional data with high sparsity like this data set (sparsity = 74%) (28). OTU tables were filtered to OTUs present in at least five samples with >10 reads per OTU (sparsity = 53%). For the Co-Net based analysis, the OTU relative abundances were calculated, and the obtained OTU tables were transformed [log_10_(*x* + 1)] before calculating Spearman correlation scores using Co-Net in Cytospace (54). The parameters included the selection of top 5% correlations (edge selection, quantile = 0.05, top and bottom) and the computing of *P* values by Fisher Z-score with multiple-test correction (Bonferroni, *P* = 0.001). For the SparCC-based analysis, the filtered OTU tables (OTU raw abundances) were used to calculate SparCC correlation scores (with default parameters). Pseudo *P* values were inferred from 1,000 bootstraps. Only correlations with *P < *0.001 were kept for further analyses. Cytoscape (v3.7.1) was used for network visualization and determination of betweenness centrality (i.e., the fraction of shortest paths passing through a given node) and closeness centrality values (i.e., the average shortest distance from given node to each other node). Node-rewiring score (Dn-score) was calculated via the DyNet package in Cytoscape (29). For each node, its connected neighbors are compared between two networks and the changes (rewiring) are quantified. Differences between conditions were tested using a nonparametric multivariate test (Dunn’s test, Bonferroni corrected, *P*_adj_ < 0.05). Microbial hubs were identified as top 5% OTUs showing maximum betweenness centrality and closeness centrality scores.

### Data availability.

Sequencing data are available under NCBI BioProject PRJNA438596. OTU tables and scripts are available (https://github.com/IshtarMM/Dynamic_LeafMicrobiome). All A. thaliana accession numbers used in this study have been published previously, and seeds are available from stock centers.
