# Supplementary figures and images for: The Leaf Microbiome of Arabidopsis Displays Reproducible Dynamics and Patterns throughout the Growing Season
Source: mBio. 2022 Apr 14;13(3):e02825-21. doi: 10.1128/mbio.02825-21 (PMC9239250; doi:10.1128/mbio.02825-21)

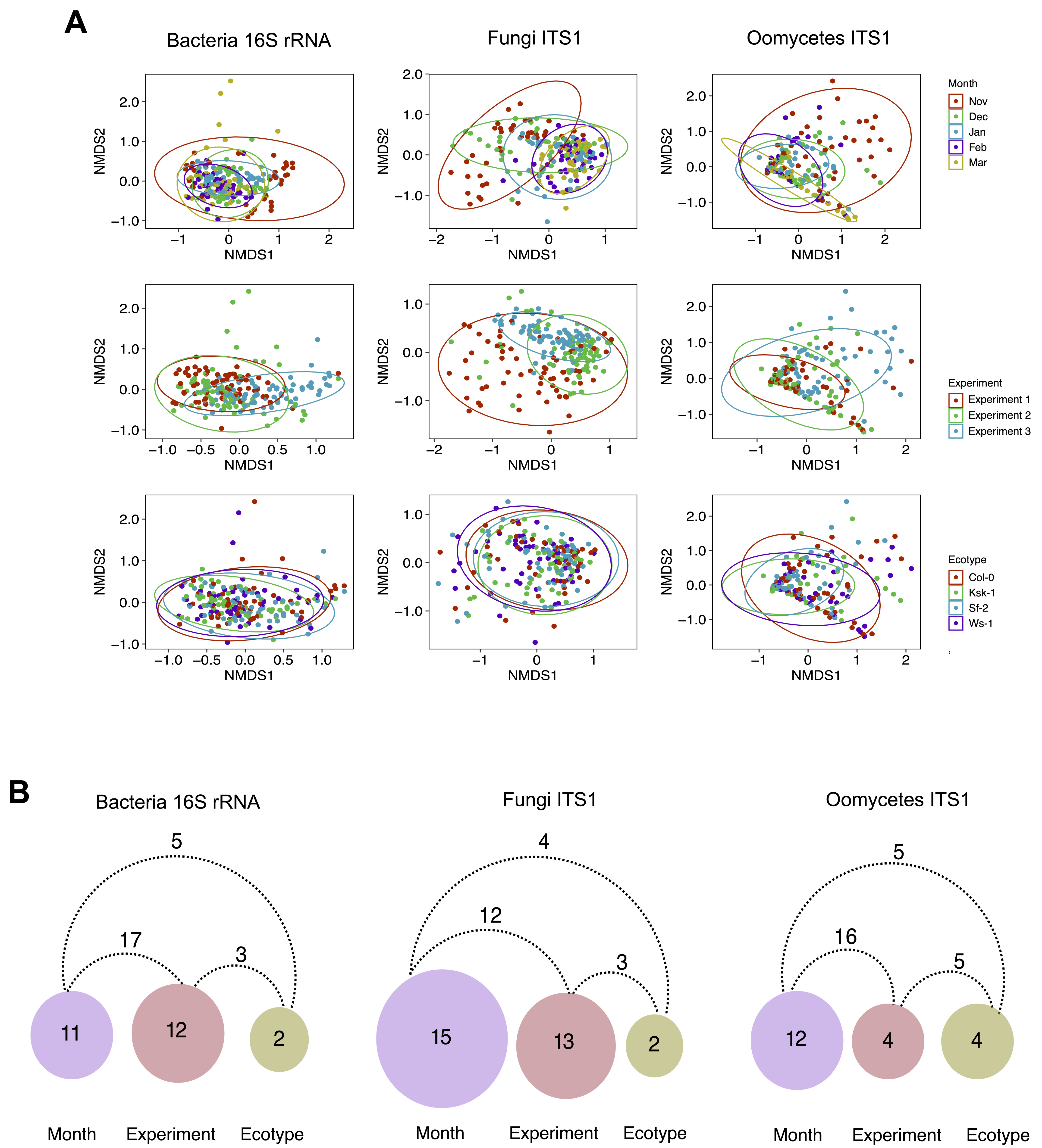

Supplement: FIG S1 [file mbio.02825-21-s0001.tif]

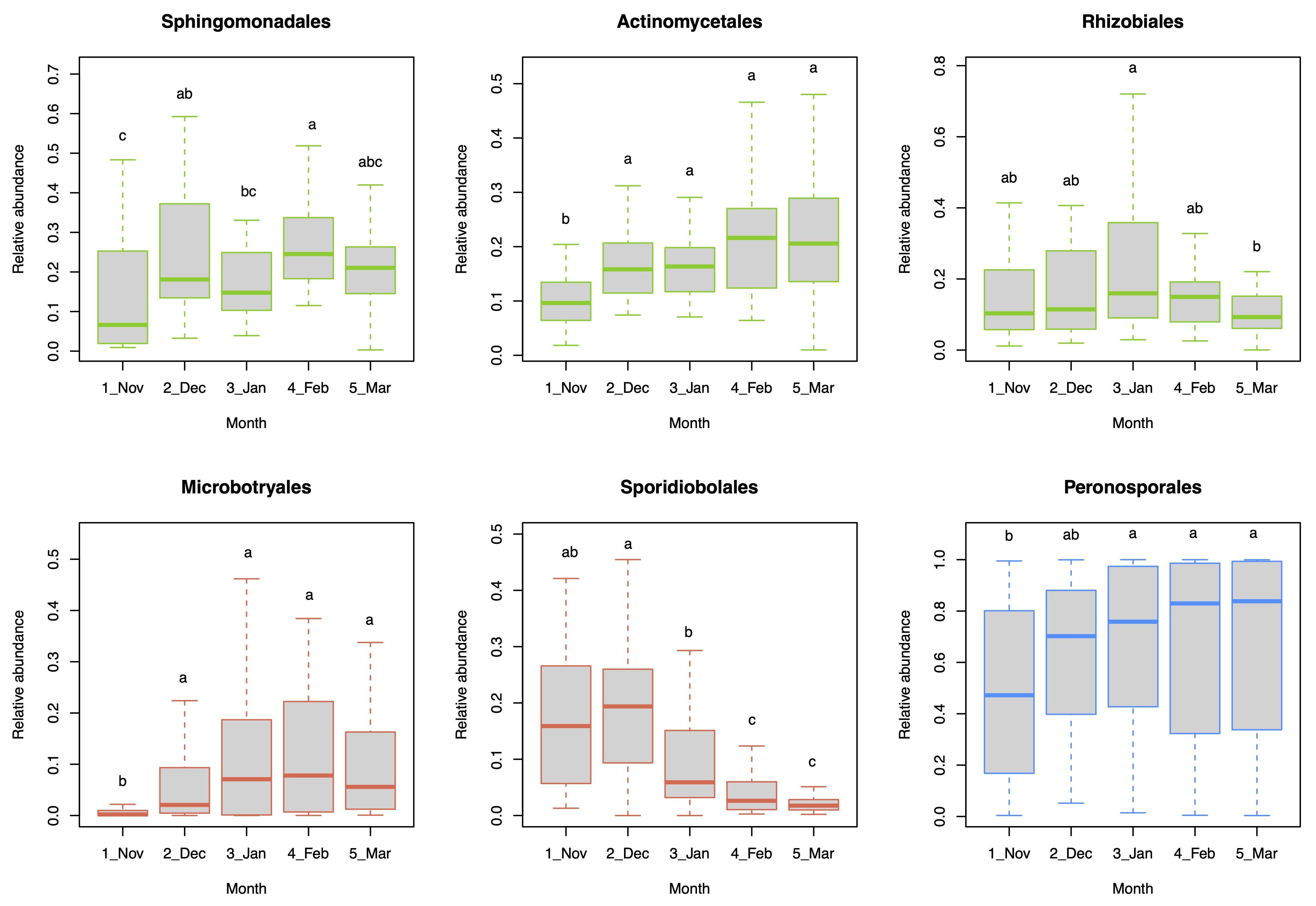

Supplement: FIG S2 [file mbio.02825-21-s0002.tif]

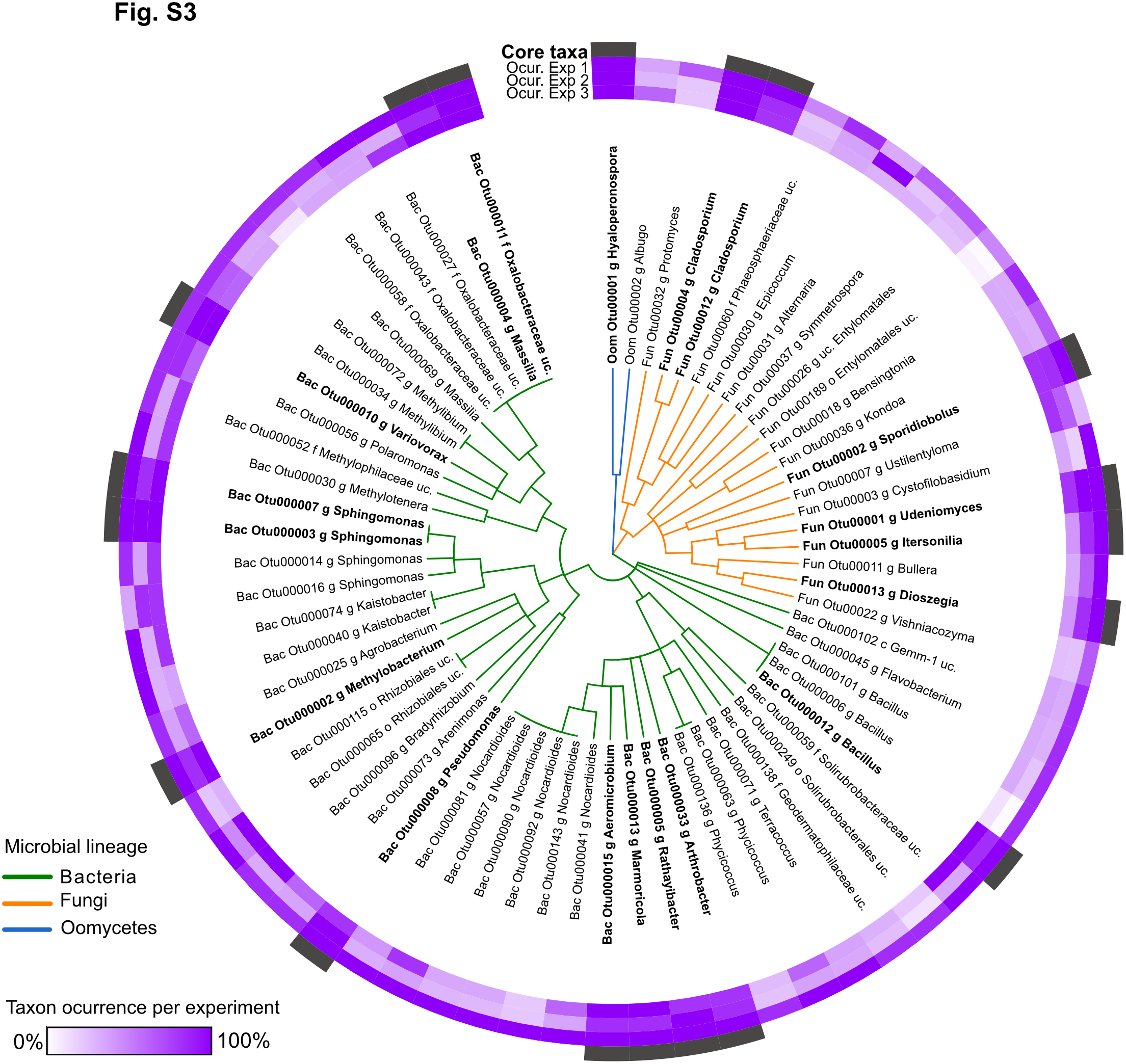

Supplement: FIG S3 [file mbio.02825-21-s0003.tif]

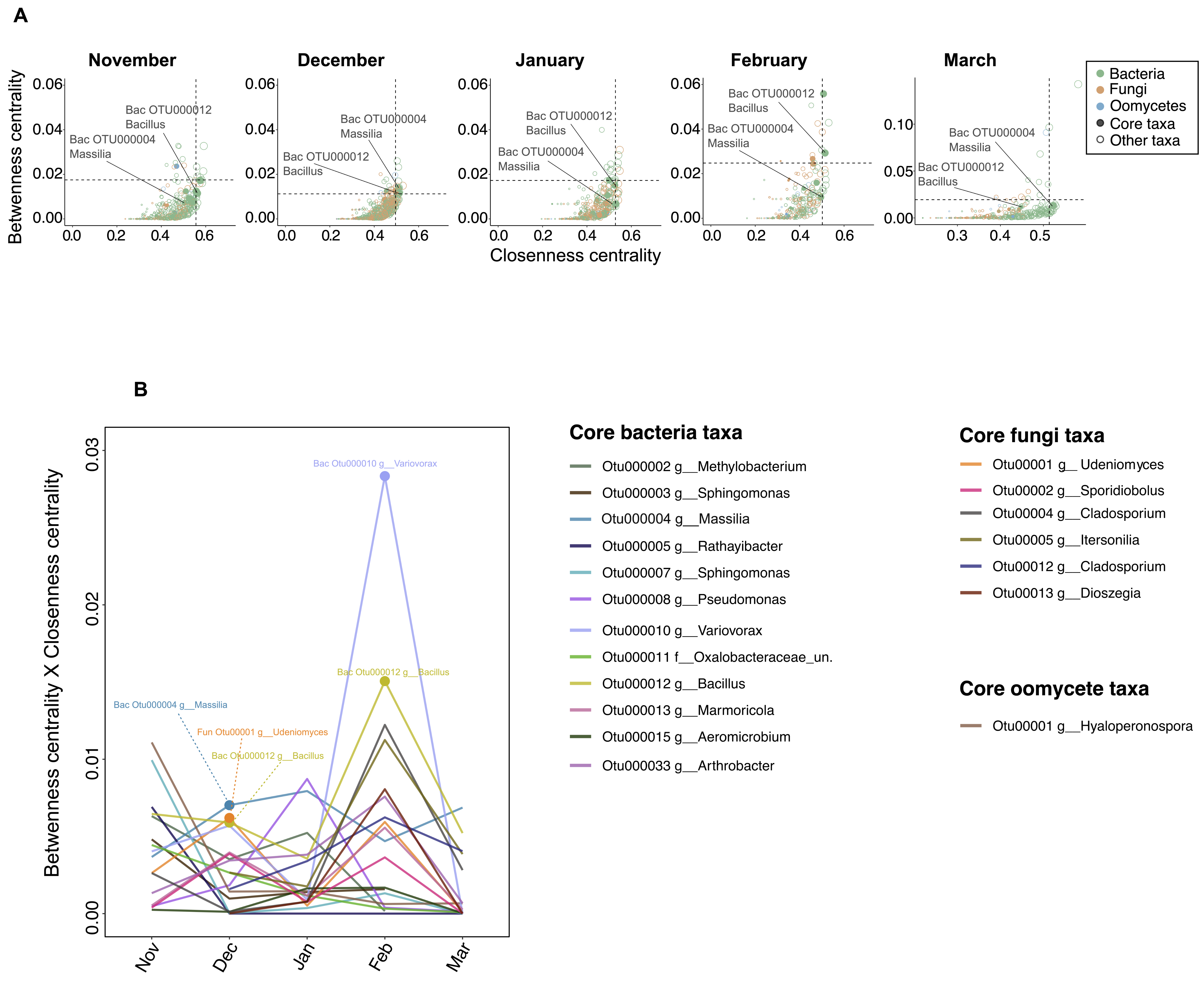

Supplement: FIG S4 [file mbio.02825-21-s0004.tif]
